# The “What” of Athletes’ Goal Pursuit and Its Relationships to Goal-Related Processes and Well- and Ill-Being

**DOI:** 10.3390/bs15050661

**Published:** 2025-05-12

**Authors:** Natalia Martínez-González, Francisco L. Atienza, Joan L. Duda, Isabel Balaguer

**Affiliations:** 1 Department of Social Psychology, Faculty of Psychology and Speech Therapy, University of Valencia, 46010 Valencia, Spain; natalia.martinez@uv.es; 2 Department of Personality, Evaluation and Psychological Treatment, Faculty of Psychology and Speech Therapy, University of Valencia, 46010 Valencia, Spain; francisco.l.atienza@uv.es; 3 School of Sport, Exercise and Rehabilitation Sciences, University of Birmingham, Birmingham B15 2TT, UK; j.l.duda@bham.ac.uk

**Keywords:** goal content, self-determination, goal motives, intrinsic goal, extrinsic goal

## Abstract

Goal Contents Theory (GCT) postulates that the goals people pursue not only direct their perceptions of and behaviors relevant to goal pursuit, but also hold implications for their well- and ill-being. Extending past sport work grounded in this theory, this study explored athletes’ self-generated goals and examined whether these goals were differentially aligned with goal-related processes and well- and ill-being. A total of 414 university team athletes (206 women and 208 men) completed a questionnaire at the beginning of the sport season. The results showed that intrinsic goals, assessed in an open-ended format, were more heterogeneous in terms of content, and more common among athletes than extrinsic goals. In addition, women reported more intrinsic goals and fewer extrinsic goals than men. MANCOVA revealed that athletes who pursued extrinsic goals reported significantly lower self-efficacy and greater perceptions of goal difficulty than athletes with intrinsic goals. No differences in goal motives and well- and ill-being indicators emerged. Finally, two models were tested that illustrate how goal content is related to self-efficacy for goal attainment, goal motives, and well- and ill-being. Overall, the findings were largely congruent with GCT and indicate that the quality of athletes’ goal-related processes and their well- and ill-being vary as a function of whether they are pursuing intrinsic or extrinsic goals.

## 1. Introduction

The motivation literature recognizes that goals are crucial as instigators and directors of human action (Ryan & Deci, 2000). From this perspective, understanding not only “why” individuals pursue specific goals but also “what” particular goals they set is essential. Both factors are crucial for understanding goals’ effectiveness, persistence, and their impact on well-being (Ryan et al., 1996). In sports, identifying the specific goals that athletes pursue can assist professionals in gaining a more nuanced and practical knowledge regarding variability in their daily practice and progress throughout the season.

Grounded in Self-Determination Theory (SDT; Deci & Ryan, 1985, 2000; Ryan & Deci, 2000, 2017) and comprising one of the six mini-theories within SDT, the Goal Contents Theory (GCT; Kasser & Ryan, 1993, 1996) holds that the type of goals that a person pursues has implications for their personal and relational functioning. It is suggested that the examination of goal content can provide a better understanding of the functioning of people in different contexts (Deci & Ryan, 2000; Kasser & Ryan, 1993, 1996). This theory focuses on the specific contents of people’s goals; “what” their behavior is directed towards and “what” is the goal of their activity (Ryan & Deci, 2000). This should not be confused with the “why” of behavior; that is, the reasons that motivate the person to carry out the behavior or what regulates goal striving, which may be more or less self-determined (Lens & Vansteenkiste, 2006; Ryan & Deci, 2017).

Regarding goal content, goals can be classified as intrinsic or extrinsic, each having different implications for people’s health and functioning (Deci & Ryan, 2000; Ryan & Deci, 2000). While intrinsic goals align with innate human growth tendencies, extrinsic goals focus on obtaining external rewards or social recognition (Deci & Ryan, 2000; Ryan & Deci, 2017). Common intrinsic goals include personal growth (self-acceptance and development), affiliation (having deep, enduring relationships), community contribution (generativity and helping others), and physical health (to feel healthy and free of illness). In contrast, extrinsic goals include financial success (being wealthy and materially successful), popularity (being famous or admired), and having an appealing image (being physically attractive) (e.g., Kasser & Ryan, 1996; Niemiec et al., 2009). Recent research has expanded this classical classification to include self-expression (to pursue authentic and volitional interests and passions) and mastery (seeking to develop high-level skills and capabilities) as intrinsic goals, and power (control and dominance over others) and social adherence (to fit in and not be excluded) as extrinsic goals (Martela et al., 2019).

Given these differences in their content, it has been postulated that intrinsic goals, contrary to extrinsic goals, promote adaptive outcomes for a person’s development (Sheldon et al., 2004). There is considerable evidence that both pursuing and achieving intrinsic goals are positively related to well-being indicators. In contrast, extrinsic goals not only fail to correlate with well-being but have, in some cases, been associated with indicators of ill-being (e.g., Bradshaw et al., 2018; Kasser & Ryan, 1993, 1996; Niemiec et al., 2009; Romero et al., 2012). Studies have shown that, in one’s life in general, prioritizing the pursuit of intrinsic goals instead of extrinsic goals was related to high levels of vitality and life satisfaction (Kasser & Ryan, 1993; Romero et al., 2012) and low levels of anxiety and depression (Kasser, 2002; Schmuck et al., 2000). In contrast, a relatively strong focus on extrinsic goals has been associated with lower subjective vitality (Kasser & Ryan, 1996), more physical and emotional symptoms (Unanue et al., 2014), and engagement in high-risk behaviors, such as drug consumption (Kasser & Ryan, 2001; Williams et al., 2000).

GCT has been successfully tested not only in a wide variety of cultures (e.g., Grouzet et al., 2005; Unanue et al., 2014) and amongst varied ages (e.g., Easterbrook et al., 2014), but also in different domains, such as education (e.g., Vansteenkiste et al., 2004a, 2004b), work (e.g., Vansteenkiste et al., 2007a), and exercise (e.g., Sebire et al., 2009; Sicilia et al., 2017). In the latter setting, the promotion of intrinsic goals fostered both the quantity and the quality of exercise persistence, as well as enjoyment, effort expenditure, and performance (Vansteenkiste et al., 2004a, 2004c). Conversely, the promotion of extrinsic goals has been associated with short-term but not long-term persistence, and a decrease in individuals’ enjoyment and performance (Vansteenkiste et al., 2007b).

Despite the development of the Goal Content for Exercise Questionnaire (CGEQ; Sebire et al., 2008) to assess goal content in exercise, there is no version specifically adapted to the sports context. In addition to this limitation, quantitative methods for studying goals impose constraints by limiting the depth of information participants can provide about their goal pursuits and potentially inducing a ‘now that you mention it’ effect, where responses are shaped by pre-set items (Urdan & Mestas, 2006). To address these issues, the present research has focused on self-generated goals, allowing athletes to express their goals in their own words. The significance of self-generated goals lies in the fact that individuals create them independently, making them more consciously salient in their lives (Sheldon & Elliot, 1999).

Although several studies have examined self-generated goals in athletes, none have done so from the perspective of GCT. Prior research primarily applied the self-concordance model (SCM; Sheldon & Elliot, 1999), which focuses on the “why” of goal pursuit rather than the “what”. SCM-based research has consistently shown that autonomous goal motives (aligned with personal values) promote well-being and goal progress, whereas controlled motives (driven by external pressure or guilt) compromise athletes’ functioning and well-being (Gaudreau & Braaten, 2016; Healy et al., 2014; Smith et al., 2007, 2010, 2011). Considering this research gap, the aim of this study is to analyze the content of the goals that athletes set for themselves during sports practice from the GCT perspective and explore the implications of pursuing primarily intrinsic or extrinsic goals for athletes’ motivation and well-being.

Past research suggests that intrinsic goals are mostly pursued for autonomous motives, so that the person tends to experience them as volitional and feels that they emanate from and as an expression of the self (e.g., Ryan & Deci, 2017; Sebire et al., 2008; Sheldon et al., 2004). Accordingly, previous studies have shown that intrinsic goals are more likely to be pursued because they are inherently satisfying, promote enjoyment, and are more in accordance with individual values and interests (Ryan & Deci, 2017). This facilitates the commitment individuals have to these goals, as well as their perception of self-efficacy to achieve them. Conversely, extrinsic goals are pursued primarily for controlled reasons, which generate in the person a feeling of pressure or coercion to act (e.g., Sheldon et al., 2004; Sebire et al., 2008). Thus, extrinsic goals promote a lower-quality persistence, which is less stable and is apparently focused on the anticipated extrinsic outcomes (Vansteenkiste et al., 2004b). In these cases, individuals may experience feelings of inefficacy and incompetence due to their inability to persist in the desired behaviors (Vansteenkiste et al., 2007b).

Regarding well- and ill-being, evidence suggests that placing more importance on intrinsic goals rather than extrinsic ones during physical exercise is positively associated with autonomous motivation (e.g., Gunnell et al., 2014; Sebire et al., 2011), enjoyment (Vansteenkiste et al., 2004a, 2004c), and subjective vitality (Gunnell et al., 2014). In contrast, pursuing extrinsic goals reduces enjoyment (Vansteenkiste et al., 2004a, 2004c).

Finally, an additional aim of this study is to examine the potential differences between men and women in goal content among athletes. Although previous research has not applied GCT to assess self-generated goals in this population, existing evidence suggests that men are more likely to prioritize extrinsic life goals, such as wealth and fame, compared to women (Raimundi et al., 2019; Romero et al., 2012). In the sports domain, men tend to score higher in competitiveness and win orientation (Gill et al., 1996), and are more focused on demonstrating their abilities in achievement contexts (Castillo et al., 2000; Moreno et al., 2008; White & Duda, 1994). In contrast, women are more inclined toward task-oriented goals, perceiving competence through effort, learning, and task mastery (Hanrahan & Biddle, 2002; Hanrahan & Cerin, 2009).

In sum, the objectives of the present study are as follows: (1) To analyze the goal content of self-generated goals that athletes pursue during their competitive season, applying GCT. (2) Examine differences in goal content between men and women. (3) Examine differences according to the type of goals in relation to other relevant goal-related variables (self-efficacy for goal attainment, goal motives, difficulty, directed effort, and controllability) and well- and ill-being. And finally (4) test two integrative models examining the relationships between athletes’ self-generated goal content (‘what’) and self-efficacy for goal attainment and goal motives (‘why’), and their implications for well- and ill-being.

Based on these objectives, the following hypotheses were formulated: (1) Women will report more intrinsic and fewer extrinsic goals than men. (2) Intrinsic goals will be positively associated with variables that facilitate optimal functioning (e.g., self-efficacy, autonomous goal motives, goal controllability, and vitality), whereas extrinsic goals will be associated with indicators of compromised functioning (e.g., controlled goal motives, goal uncontrollability, and physical and emotional exhaustion). (3) Goal content (intrinsic or extrinsic) will predict well- and ill-being outcomes through its effect on athletes’ self-efficacy and goal motives (mediators). Specifically, intrinsic goal content will positively predict vitality and negatively predict physical and emotional exhaustion, through its positive effect on self-efficacy and autonomous goal motives. In contrast, extrinsic goal content will positively predict exhaustion and negatively predict vitality, through its effect on lower self-efficacy and more controlled goal motives.

## 2. Materials and Methods

### 2.1. Participants

Three Valencian universities (Valencia, Spain) that competed in the annual Regional University Sports Championship (CADU) participated in the study. The total sample consisted of 414 university team athletes (206 women and 208 men) between 17 and 33 years (*M* = 20.61, *SD* = 2.58) who completed the provided questionnaires at the beginning of the sports season. Basketball, handball, football, indoor football, rugby, and volleyball were the sports involved.

All participants were selected to represent their university teams in official competitions. The CADU serves as the first stage of the university sports competition system in Spain: teams first compete at the regional level, and those who qualify advance to the national university championship. The best-performing teams at the national level may then represent Spain in the European University Championships. On average, athletes reported training 1.8 ± 0.78 h per week.

### 2.2. Procedure

The research was presented in each university sports service to request participation, where in addition, the study procedure was reviewed by the corresponding Human Research Ethics Committee. Once approval was obtained, trained researchers proceeded with data collection at the beginning of the sports season. First, they informed the athletes about the confidentiality and anonymity of their answers, as well as about the voluntary nature of participation. Then, all the athletes who agreed signed a consent form to join the research. Finally, a package of questionnaires was administered, adapted in each case to refer to men or women (due to the specific demands of the Spanish language).

### 2.3. Measures

Self-generated goals: First, the athletes were instructed to read the following definition of goal: “objectives you are striving to achieve in your university sport during the current season”. Then, based on the idiographic methodology proposed by Sheldon (2002) to capture self-generated goals, they were asked to write a response to the following statement “identify your most important sporting goal that you hope to make progress on during the current season”. Similar procedures have been used previously in the framework of the self-concordance model (Sheldon & Elliot, 1999) and in the sporting context (e.g., Smith et al., 2007, 2011; Smith & Ntoumanis, 2014).

Goal-related variables: In addition to their self-generated goals, athletes had to respond to one section that measured different aspects related to their goals. Specifically, the perception of self-efficacy to achieve the goal, goal motives, goal-directed effort, and the perception of goal difficulty were assessed in an ad hoc questionnaire. Self-efficacy for goal attainment was measured using 1 item (i.e., “To what extent do you feel capable of achieving this goal?”) that has been previously used in previous Spanish research (Arruza et al., 1998; Sansinenea et al., 2008). Regarding goal motives, goal-directed effort, and goal difficulty, items were adapted for this research and translated into Spanish from previous studies in sports goals (Healy et al., 2014; Smith et al., 2007, 2011). Specifically, the motivation underlying their goals was measured in terms of whether athletes were pursuing their goal for autonomous (4 items, e.g., “Because of the fun and enjoyment the goal provides me”) or controlled (4 items, e.g., “Because someone else wants me to”) reasons. Then, 3 items were used to assess goal-directed effort (e.g., “Rate the amount of effort you are putting into this goal during the current season”) and another 3 items evaluated athletes’ perception of goal difficulty (e.g., “Rate how challenging your goal is for you”). For all these variables, participants responded on a Likert scale ranging from 1 (not at all) to 7 (very much so). Goal motives, goal-directed effort, and goal difficulty scales have shown adequate validity and reliability in previous studies with athletes (Healy et al., 2014; Smith et al., 2007, 2011). Moreover, initial data on Spanish athletes have shown acceptable values in this regard (Martínez-González et al., 2021a, 2021b), with Cronbach’s alpha coefficients ranging from 0.70 to 0.73.

Well-being: Based on the previous literature (e.g., Ryan & Deci, 2001), subjective vitality was employed as a eudaimonic well-being indicator. Thus, the Spanish version of the Subjective Vitality Scale (SVS; Ryan & Frederick, 1997) was applied (Castillo et al., 2017) for the measurement of athletes’ “experience of having positive energy available to or within the regulatory control of one’s self” (Ryan & Frederick, 1997, p. 530). This scale, made up of 6 items (e.g., “I feel alive and vital”), was answered using a 7-point Likert scale from 1 (not at all true) to 7 (very true). The Spanish version of the scale has shown adequate reliability and validity in sporting contexts (e.g., González et al., 2015; Pineda-Espejel et al., 2023; Tristán et al., 2021), with internal consistency coefficients ranging from 0.85 to 0.89.

Ill-being: The subscale of physical and emotional exhaustion from the Athlete Burnout Questionnaire (ABQ; Raedeke & Smith, 2001) was used as an ill-being indicator. For this purpose, the Spanish version of the subscale was applied (Balaguer et al., 2011), composed of 5 items (e.g., “I am exhausted by mental and physical demands of sport”) that were rated on a Likert scale from 1 (almost never) to 5 (almost always). This subscale has also demonstrated good psychometric properties in previous studies with Spanish athletes, showing an internal consistency of 0.83 (e.g., Castillo et al., 2010).

### 2.4. Data Analysis

In this research, qualitative and quantitative data were collected with the aim of answering the research questions. Generally, analyses begin with the processing of qualitative data, following several stages: material sourcing and transcription (stages 1 and 2), unitization (stage 3), categorization (stage 4), and coding (stage 5). As a result of these steps, coded data can be used for subsequent quantitative (descriptive, exploratory, and hypothesis testing) analyses. In the present study, a content analysis of the athletes’ goals (qualitative material) was carried out first, followed by the assignment of numerical values to the different categories for further analyses.

#### 2.4.1. Goal Content Analysis

In this first phase of the analysis process, athletes’ self-generated goal responses were analyzed. Due to the written nature of data collection, the first two stages typical in this process (material sourcing and transcription) do not apply. Therefore, to examine responses and classify them into content analytical units (categories or codes), the analysis began with stage 3 of unitization and stage 4 of categorization, which correspond with the content analysis. In developing the category scheme, a deductive approach was conducted, so the existing theory and theoretical concepts guided the categorization process and provided the rationale for establishing “standard categories”. Once it was established, in stage 5, instructions for the coding process were elaborated, detailing the rules, definitions, and examples that would guide the coding. Finally, three recruited experts with knowledge and experience in the theoretical framework received the material (the instructions and the dataset) and conducted the coding process, where each coding unit was assigned a main and a subcategory code.

As a measure or inter-rater agreement, the statistic Fleiss’ kappa (Fleiss, 1971; Fleiss et al., 2003) was used to determine the level of agreement between the three judges. This procedure is recommended when the method of assessment, as in this case, is measured on a categorical scale. The Fleiss’ kappa values were interpreted following the guidelines from Landis and Koch (1977) that classified the values as poor (<0.00), slight (0.00–0.20), fair (0.21–0.40), moderate (0.41–0.60), substantial (0.61–0.80), and almost perfect (0.81–1.00). Once it was verified that the percentages of agreement were high and acceptable, analysis of the data started (see Section 3).

#### 2.4.2. Analysis of Differences and Association Between Variables

After analyzing the goal content, the IBM SPSS Statistics 25 software was used to carry out quantitative analyses according to objective 2 (examine differences between men and women in goal content) and objective 3 (explore goal associations with other relevant goal-related variables and with athletes’ well- and ill-being). Prior to the main analyses, the dataset was prepared and qualitative data on goals were transformed into quantitative data. Once the database was ready, preliminary analyses were conducted. Then, in line with the second objective, Chi-square tests of independence were performed to examine the differences in categories and controllability by athletes’ gender. In addition, this test was employed to study the relationship between categories and controllability. To measure the effect sizes, the coefficient Phi (φ) was estimated, considering a value of 0.10 a small effect, 0.30 a medium effect, and 0.50 or higher a large effect. Additionally, with regard to the third objective, a between-subjects multivariate analysis of covariance (MANCOVA) was performed, controlling for gender on seven dependent variables: self-efficacy for goal attainment, autonomous goal motives, controlled goal motives, goal difficulty, goal-directed effort, vitality, and physical and emotional exhaustion. The independent variable was the type of category (intrinsic or extrinsic). This technique of analysis is recommended for exploring the relationships between different dependent variables at each level of the independent variable, and in addition, it offers some control over the overall type I error rate (Tabachnick et al., 2007). Prior to the main analyses, assumptions of normality, the homogeneity of variance–covariance matrices, linearity, and multicollinearity were evaluated. Then, all multivariate effects were evaluated and partial η^2^ was used to estimate the effect sizes. In this regard, Cohen’s (1988) suggestions to interpret the values were followed, considering small, medium, and large effects values of 0.01, 0.06, and 0.14, respectively.

Finally, mediation analyses with a dichotomous antecedent (model 81, with 10,000 bootstrapping to generate 95% confidence intervals via the percentile method) were performed using Process macro, version 4.0 for SPSS (Hayes, 2022), to address the fourth objective. The independent variable was coded as a dummy variable (extrinsic goal = 0 and intrinsic goal = 1) in a procedure that examined both the direct and indirect effects of athletes’ goal content on their vitality and on their physical and emotional exhaustion. In the case of indirect effects, following the theoretical postulates, self-efficacy was included in the model as the primary mediator, and goal motives as the secondary mediators.

## 3. Results

### 3.1. Athletes’ Goal Content

#### 3.1.1. Category Scheme and Intercoder Reliability Measures

The final scheme comprised three categories for coding the goal based on the type of content (intrinsic, extrinsic, and ambiguous), two to code the goal controllability (controllable or uncontrollable), and some subcategories. Regarding the main categories, it should be noted that the ambiguous category was introduced following the recommendations of Sebire et al. (2008) about some goals that a priori are difficult to classify in terms of intrinsic–extrinsic goal content differentiation. One example is the goal of “losing weight through exercising”, which can be related to maintaining good health (intrinsic) or to improving one’s appearance (extrinsic). Each intrinsic goal was classified into one of the seven intrinsic subcategories (social affiliation, self-expression, community contribution, personal growth, mastery, psychological and physical health, or multiple), whereas each extrinsic goal was listed in one of the seven extrinsic subcategories (social adherence, financial success, image, power, social recognition/popularity, superiority, or multiple). All of these subcategories, as well as the main categories, were established based on the previous literature. They were also classified based on whether they were controllable (i.e., dependent on internal factors such as effort) or uncontrollable (i.e., dependent on external factors such as task difficulty). The definitions of the main codes and subcategories can be found in Appendix A.

Overall, the Fleiss’ kappa for both categories (κ = 0.67 (95% CI, 0.62 to 0.73), *p* < 0.001) and subcategories (κ = 0.70 (95% CI, 0.67 to 0.73), *p* < 0.001) indicated substantial agreement between the experts’ judgements, whereas it was almost perfect for controllability (κ = 0.81 (95% CI, 0.72 to 0.90), *p* < 0.001). In addition, individual Kappa analysis results (Table 1) showed levels ranging from moderate (0.49) to perfect (1.0) for categories, subcategories, and controllability, except for the “ambiguous” category, whose value was fair (0.37). Due to the fact that, after a review, the data obtained in the ambiguous category did not reach a moderate level, it was decided not to analyze this category further (this was not considered for the analyses of differences and associations between variables). It should be noted that the extrinsic goal subcategories of “social adherence”, “image”, “power”, and “superiority” are not represented, as the judges did not find any goals in the sample that fit these definitions.

#### 3.1.2. Distribution of Goal Content Categories and Subcategories

The frequency distribution and percentages of categories, subcategories, and controllability are presented in Table 1. With regard to categories, a higher number of the total goals analyzed were classified as intrinsic goals than extrinsic goals (61.8% vs. 31.4%). Representative examples of intrinsic goals were pursuing the goal “to have a good time/to enjoy/to have fun”, “to improve (in my position, as a player, my game, my technique, my skills…)”, and “to learn (new techniques, from my teammates, how to move better)”. Examples of extrinsic goals include athletes pursuing the goal for reasons such as “to win the competition”, “to qualify”, and “to be in the top three at the regional level”.

Concerning the subcategories of intrinsic goals, goals that mixed two or more intrinsic reasons were found in the first place, followed by the mastery subcategory. Also notable but less represented were self-expression, social affiliation, and the rest of the intrinsic goal subcategories. Upon analyzing the “multiple subcategory”, it was found that 41.57% of these goals mixed “personal growth” and “mastery” (e.g., “to learn from my mistakes and improve”). Among others, some of the combinations that were found to a lesser extent were “social affiliation”, “personal growth”, and “mastery” (e.g., “to know how to work and get on with colleagues”) or “social affiliation” and “mastery” (e.g., “to improve as a team”).

Examples found of the rest of the intrinsic subcategories are listed below:

- Mastery: “to improve (in my position, as a player, my game, my technique, my skills…)”, “to learn (new techniques, from my teammates, to move better)”, or “to increase my level as athlete”;
- Self-expression: “to have a good time/to enjoy/to have fun”, “to play”, or “to practice a sport that I like/I am passionate about/I have fun with”;
- Social affiliation: “to get to know people”, “to get a united team/team building”, or “to make friends”;
- Psychological and physical health: “to keep fit”, “to practice sport for health”, or “to get back to pre-injury fitness levels”;
- Personal growth: “to trust myself more when I play”, “to overcome my insecurities”, or “to grow as a person”;
- Community contribution: “to bring good things to the team”.

With reference to extrinsic subcategories, goals that were pursued for more than one extrinsic reason were the most frequent. In the remaining subcategories, only examples of “financial success” (e.g., “to obtain university credits”) and “social recognition/popularity” (e.g., “to represent the university”) were found in the sample. The “multiple” subcategory comprised 96.5% of goals that combined “social recognition”, “popularity”, and “superiority” (e.g., “to win against another university”), while the remaining ones included the previous ones and added “power” (e.g., “to be a starter in my team”).

Finally, 58.2% of the goals were classified as uncontrollable and 41.8% as controllable. Examples of uncontrollable goals were “to win the competition” and “to qualify”, whereas controllable goals were for example “to improve (in my position, as a player, my game, my technique, my skills…)” and “to learn (new techniques, from my teammates, how to move better)”.

### 3.2. Descriptive Findings, Goal Differences, and Association Between Variables

#### 3.2.1. Preliminary and Descriptive Analyses

The analysis of outliers identified five participants who were removed from the sample due to extreme values. Descriptive statistics, reliability, and bivariate correlations are presented in Table 2. Mean scores revealed that athletes reported high perceptions of self-efficacy and goal-directed effort, and medium-high of goal difficulty. Also, they showed high values for autonomous goal motives and medium-high values for vitality, whereas controlled goal motives and physical and emotional exhaustion were low. Regarding reliability, Cronbach’s alpha coefficients showed acceptable and satisfactory values for the scales with alphas ranging from 0.70 to 0.91.

According to the bivariate correlations, the results indicated that athletes’ self-efficacy was positively associated with autonomous goal motives and goal-directed effort and negatively with perceived goal difficulty. However, athletes’ self-efficacy for goal attainment was not significantly related to pursuing goals for controlled reasons. Additionally, only autonomous goal motives (but not controlled ones) were positively and significantly related to the effort directed toward goal achievement.

In terms of well-being and ill-being, perceived self-efficacy, autonomous goal motives, and goal-directed effort were both positively associated with vitality and negatively associated with physical and emotional exhaustion. In contrast, controlled goal motives were not related to vitality, but were positively related to physical and emotional exhaustion. Consistent with the previous literature, the relationship between autonomous and controlled goal motives, as well as between well- and ill-being indicators, was significant and negative (e.g., Martínez-González et al., 2021b).

#### 3.2.2. Differences Between Men and Women

The results revealed significant differences according to gender in the goal categories (χ^2^ = 10.31, df = 1, *p* < 0.01, φ = 0.16) and in goal controllability (χ^2^ = 14.89, df = 1, *p* < 0.01, φ = 0.21). Regarding categories, findings showed that women reported more intrinsic and fewer extrinsic goals than men. Specifically, 73.8% of women’s goals were classified as intrinsic and the remaining 26.2% as extrinsic, whereas only 58.5% of men’s goals were intrinsic and 41.5% were extrinsic. Regarding controllability, 51.9% of women’s goals were classified as controllable and 48.1% uncontrollable, whereas in men controllable goals were 32.1% of the total goals and 67.9% were uncontrollable. These results showed that women pursued more controllable and fewer uncontrollable goals than men. Effect sizes in both cases were small.

#### 3.2.3. Categories and Controllability

The findings revealed significant differences in goal categories depending on controllability (χ^2^ = 136.08, df = 1, *p* < 0.05, φ = 0.59), with intrinsic goals classified as controllable to a greater extent compared with extrinsic goals. The results showed that intrinsic goals were 63.3% controllable and 36.7% uncontrollable, whereas extrinsic goals were only 2.8% controllable and 97.2% uncontrollable. The effect size was large.

#### 3.2.4. MANCOVA

The Box’s M of 77.07 indicated that the homogeneity of covariance matrices across groups was not assumed (F(28, 247691.39) = 2.69, *p* =.001). Hence, a Pillai’s trace was employed, as it is argued to be the most robust statistic for general protection against departures from the multivariate normality and homogeneity of variance–covariance matrices (Tabachnick et al., 2007). With the use of Pillai’s criterion, the combined DVs were significantly different by type of category (Pillai’s Trace = 0.10, F (7, 377) = 5.95, *p* = 0.001, partial η^2^ = 0.10) after controlling for gender.

To investigate the impact of each effect on individual DVs, a univariate F-test using an alpha level of 0.05 was performed (Table 3). The main effects of the category were significant for self-efficacy, for which the partial eta squared indicated small effects, and goal difficulty, for which the effect was medium. Pair-wise comparison indicates that the perception of goal difficulty was higher in the extrinsic category, whereas self-efficacy for goal attainment was higher in the intrinsic one. There were no significant differences according to the type of goal category on athletes’ goal motives, goal-directed effort, or well- and ill-being indicators.

#### 3.2.5. Mediation Models

**Figure 1:**
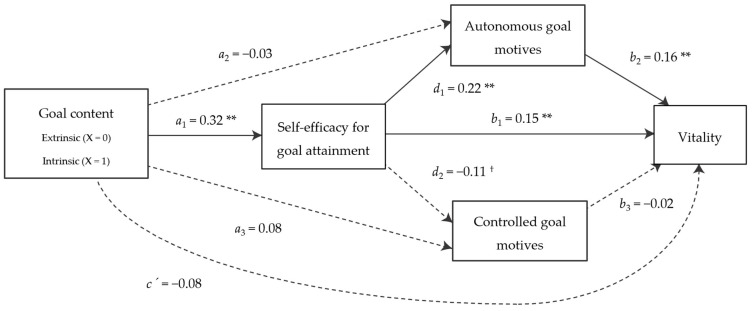
Mediation model of the associations between goal content, self-efficacy for goal attainment, goal motives, and vitality. Dotted arrows represent non-significant paths. ** *p* < 0.01. ^†^ *p* < 0.10.

**Figure 2:**
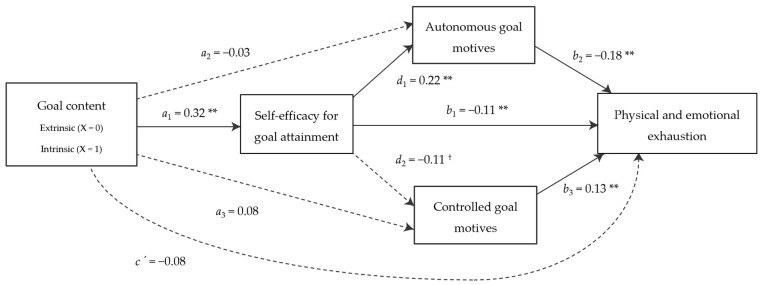
Mediation model of the associations between goal content, self-efficacy for goal attainment, goal motives, and physical and emotional exhaustion. Dotted arrows represent non-significant paths. ** *p* < 0.01. ^†^ *p* < 0.10.

According to mediation analysis, models (Figure 1 and Figure 2) revealed that intrinsic goals, in contrast to extrinsic goals, led athletes to feel more self-efficacy in achieving these goals (*B* = 0.32, *p* < 0.01), and that this in turn was associated with more autonomous motives to pursue these goals (*B* = 0.22, *p* < 0.01), as well as greater vitality (*B* = 0.15, *p* < 0.01) and lower physical and emotional exhaustion (*B* = −0.11, *p* < 0.01). Also, higher autonomous goal motives were positively related to athletes’ vitality (*B* = 0.16, *p* < 0.01) and negatively related to athletes’ physical and emotional exhaustion (*B* = −0.18, *p* < 0.01). However, athletes’ self-efficacy was not significantly related to controlled goal motives (although this relationship was negative and marginally significant, *B* = −0.11, *p* = 0.09). Goal content did not predict dependent variables either directly or only via goal motives.

A total of four statistically significant indirect effects of goal content were found on the athletes’ well- and ill-being. According to vitality, one specific indirect effect was observed through self-efficacy (IE = 0.06; 95% CI [0.01, 0.11]) and another one through the serial mediation of self-efficacy and autonomous goal motives (IE = 0.03; 95% CI [0.01, 0.03]). The indirect effects through autonomous goal motives (IE = −0.01; 95% CI [−0.04, 0.03]) and controlled goal motives (IE = −0.01; 95% CI [−0.02, 0.01]) were not significant, nor was the indirect effect through the serial mediation of self-efficacy and controlled goal motives (IE = 0.01; 95% CI [−0.01, 0.01]). With respect to physical and emotional exhaustion, indirect effects through self-efficacy (IE = −0.04; 95% CI [−0.10, −0.01]) and via the serial mediation of self-efficacy and autonomous goal motives (IE = −0.03; 95% CI [−0.03, −0.01]) were also significant. Finally, goal content did not have a significant indirect effect on physical and emotional exhaustion either via autonomous (IE = 0.01; 95% CI [−0.04, 0.04]) or controlled (IE = 0.01; 95% CI [−0.03, 0.06]) goal motives, or via the serial mediation of self-efficacy or controlled goal motives (IE = −0.01; 95% CI [−0.02, 0.01]).

## 4. Discussion

Goals, towards which people direct their behavior (Ryan & Deci, 2000), are fundamental to understanding how athletes function during their sporting experience. In this research, the well-established Goal Contents Theory (Kasser & Ryan, 1993, 1996), one of the mini-theories of the SDT, was applied to the sport context to gain a deeper knowledge of the athletes’ self-generated goal content. Due to the lack of a validated instrument for assessing the content of sporting goals from a GCT perspective, this mini-theory has not been extensively developed in this domain (Standage, 2012; Standage & Ryan, 2020). In view of the scarce previous data, this study also included analyses of differences between men and women in goal content, and an exploration of how the goals that athletes pursue relate to other goal-related processes (self-efficacy for goal attainment, goal motives, perceived goal difficulty, goal-directed effort, and controllability) and their well- and ill-being.

When addressing objective 1, once the athletes’ goals (and the other variables of interest) were collected, the first part of the goal content analysis resulted in a category scheme for coding athletes’ goals according to the type of content (3 categories and 16 subcategories) and the controllability (controllable or uncontrollable). Intercoder reliability measures in our sample indicated acceptable levels of agreement between three experts’ judgements for all except the ambiguous category, so it was decided to exclude this category and not to analyze it in more detail. The goal content distribution revealed that intrinsic goals were more frequent than extrinsic ones. In the case of intrinsic goals, a wide variety of subcategories was found, with the most numerous being multiple goals (e.g., mastery and personal growth), mastery goals, and self-expression goals. Conversely, extrinsic goals were more homogeneous in terms of content, being mainly multiple goals to do with social recognition, popularity, and superiority. With respect to controllability, a greater number of goals were found that depended on external (e.g., difficulty of the task, luck) rather than internal factors (e.g., effort).

According to the analysis of differences (objective 2), the results revealed significant differences between men and women in goal categories and controllability. Specifically, women, in contrast to men, reported more intrinsic and fewer extrinsic goals, and more controllable and fewer uncontrollable goals. These results are in line with hypothesis 1 and the previous literature, which argue that women are more focused on task-oriented goals, that is, goals related to improving, learning, or gaining mastery of a task (e.g., Hanrahan & Biddle, 2002; Hanrahan & Cerin, 2009). In contrast, men tend to be more oriented toward external rewards such as fame (e.g., Raimundi et al., 2019; Romero et al., 2012), and toward demonstrating ability and superiority over others in achievement contexts (e.g., Castillo et al., 2000; Moreno et al., 2008; White & Duda, 1994). These differences could be explained based on the socialization processes that influence gender roles, affecting the development of individuals’ goal orientations (Duda & Whitehead, 1998). On the other hand, it is also worth noting that intrinsic goals were classified to a greater extent as controllable, whereas extrinsic goals were mostly uncontrollable. These results support the theory’s statements, since intrinsic goals are, by nature, mainly goals that are under the individual’s control, such as goals to do with personal development or mastery of the task. Conversely, extrinsic goals are, by definition, goals that seek external rewards (e.g., material goods) or social recognition, so their attainment will largely be dependent on external factors (Ryan & Deci, 2017). Therefore, it is reasonable that women, who have more intrinsic goals, also have more controllable goals, while men, who have a higher percentage of extrinsic goals, also have more goals that depend on external factors.

Concerning differences in other goal-related processes according to the goal content (objective 3), results of MANCOVA revealed that athletes’ perception of self-efficacy to achieve their goals was higher in intrinsic goals, aligned with previous studies that found positive relationships between self-efficacy and goals of an intrinsic nature (e.g., Phillips & Gully, 1997). Additionally, athletes’ perceived goal difficulty was higher in extrinsic goals. This finding is in line with what is mentioned above about the nature of extrinsic goals, which tend to depend to a greater extent on external factors and are perceived as less controllable than intrinsic goals (e.g., Vansteenkiste et al., 2007b). The fact that most of these goals are not under the athletes’ control may help to explain why extrinsic goals are also perceived as difficult to achieve. However, no differences were found in terms of directed effort to obtain the goal, goal motives, or well- and ill-being indicators. With regard to directed effort, this was high for both intrinsic and extrinsic goals, showing that both can be powerful motivators in the short term, although according to the existing literature, this effort would be less stable and would even decrease over time to a greater extent for extrinsic goals (Hope et al., 2016; Vansteenkiste et al., 2004b).

On the other hand, because intrinsic goals, in contrast to extrinsic goals, are inherently satisfying, emanate from the self, and promote well-being (Ryan & Deci, 2017), we expected to find higher autonomous goal motives and subjective vitality in intrinsic goals, whereas controlled goal motives and physical and emotional exhaustion were expected to be higher in extrinsic goals. The results showed that, independently of whether the goal was intrinsic or extrinsic, athletes had high levels of autonomous goal motives and vitality, and low levels of controlled goal motives and physical and emotional exhaustion. On this point, some variables, such as perceived self-efficacy in goal attainment, may play an important role in the relationships among these variables, as can be seen in the tested model. Moreover, as the data were collected at the beginning of the season, future work may address whether there are differences when these variables are examined as the season progresses. Altogether, these findings partially support hypothesis 2. While extrinsic goals were associated with higher perceived difficulty and lower self-efficacy, no significant differences were observed in goal motives or well- and ill-being indicators, which may be influenced by other variables or temporal factors such as the timing of data collection.

Finally, two integrative models of the content of athletes’ self-generated goals (the “what”), perceived self-efficacy for goal attainment and goal motives (the “why”), and their implications for athletes’ well- and ill-being were tested in line with objective 4. These models illustrate that when an athlete pursues a goal with intrinsic content, such as “to learn from my mistakes and improve” or “to overcome my insecurities”, he or she feels more confident of being able to achieve it, which has an implication on his or her well- and ill-being via two different paths: on the one hand, because by increasing self-efficacy, well-being is promoted, and ill-being is prevented. Thus, being involved in a process of pursuing predominantly intrinsic goals, which are in line with the innate growth tendencies of the human being, makes athletes feel more effective, which in turn makes them feel more alive and vital and less physically and emotionally exhausted. On the other hand, goal self-efficacy also promotes that athletes pursue goals for autonomous reasons, i.e., because goals reflect and express the person’s internalized interests and values, which in turn promotes vitality and prevents physical and emotional exhaustion. By contrast, when an athlete pursues an extrinsic goal, considered instrumental in nature (e.g., “to win against another team” or “to qualify”), his or her perception of effectiveness in achieving this goal is lower, which implies a pursuit with fewer autonomous reasons, lower vitality, and greater physical and emotional exhaustion. Despite the fact that controlled goal motives were not significant in this mediation process, a marginally significant relationship was found between goal self-efficacy and them, suggesting that self-efficacy might also decrease goal pursuit motivated by internal or external pressures. In addition to the above, it should be noted that the relationships of goal motives with indicators of well- and ill-being are in accordance with the previous literature (e.g., Gaudreau & Braaten, 2016; Healy et al., 2014; Smith et al., 2007, 2010). These results support hypothesis 3, showing that goal content predicts well- and ill-being through its effects on self-efficacy and goal motives. Both self-efficacy for goal attainment and autonomous goal motives emerged as significant mediators. The only exception was the mediating role of controlled goal motives, which did not reach statistical significance, although a marginal association with self-efficacy was observed. This may be explained by the notion, proposed by Sezer et al. (2024), that autonomous goal motives typically mediate relationships with adaptive antecedents and well-being, whereas controlled motives are more likely to mediate maladaptive pathways linked to ill-being.

In general, these results support the tenets of the GCT and highlight the benefits of pursuing intrinsic goals over extrinsic ones. The differences in content and their ability to foster a sense of self-efficacy explain why intrinsic goals—rather than extrinsic ones—promote well-being and adaptive functioning (e.g., Sheldon et al., 2004). In this regard, the previous literature has shown that intrinsic and extrinsic goals are differently related to personal well- and ill-being (Bradshaw, 2023; Kasser, 2002; Ryan & Deci, 2017). Specifically, prioritizing the pursuit of intrinsic goals has been associated with higher levels of well-being (Kasser & Ryan, 1993; Romero et al., 2012; Zhang et al., 2019) and lower levels of ill-being (Kasser, 2002; Schmuck et al., 2000). In contrast, a stronger focus on extrinsic goals has primarily been linked to higher levels of ill-being (Unanue et al., 2014) and, in some cases, lower well-being (Bradshaw et al., 2023; Dittmar et al., 2014; Kasser & Ryan, 1996). To the best of our knowledge, prior to this study, there was no evidence supporting these claims within the sports context, much less exploring the relationships between the “what” and the “why” of the goals set by athletes (self-generated) and their consequences.

Regarding the limitations of this research, it has to be considered that it is an exploratory and cross-sectional study, so inferences of cause and effect between variables cannot be established. Future studies could collect data at different points over time in order, for example, to test the models throughout the sporting season. In addition, in this study, we focused only on the goal that athletes identified as most important, so other studies could explore more than one goal. This is one of the main limitations of research conducted with the idiographic methodology from the SCM perspective (see the systematic review by Sezer et al., 2024). Although this approach may be operational, the reality is that individuals are simultaneously working towards multiple goals (Healy, 2015; Healy et al., 2020). Moreover, results belong to a sample of young university athletes, so it would be interesting to replicate this study in order to examine goal distributions and analyze their differences in other samples and sporting contexts. It would be valuable to include athletes from different cultural backgrounds, competitive levels, and sport disciplines in order to assess the universality of the theoretical model and strengthen its external validity. Further research will also provide information to improve the procedure and facilitate decision-making, e.g., on whether the category of ambiguous goals should be maintained or redefined to ensure clearer and more consistent classification.

Overall, these findings extend the existing goal content literature in sport by taking into consideration the goals that athletes generate by themselves. Thus, the procedure described in this paper represents an advance and novelty in the study of self-reported goals in sport and it could be applied in future research to analyze the content of the goals that athletes set for themselves during the sporting season, applying a well-established theoretical framework such as the GCT. In addition, this research allowed for a deeper understanding of intrinsic and extrinsic goals in a sample of athletes. Thus, we have found that intrinsic goals were more controllable than extrinsic goals and that, through the fostering of self-efficacy for goal attainment, they promote athletes’ optimal functioning. Conversely, extrinsic goals were more uncontrollable, perceived as more difficult than intrinsic goals, and compromised functioning. In summary, the results indicate that the quality of athletes’ goal-related processes varies as a function of whether they are pursuing intrinsic or extrinsic goals, being more adaptive to pursue intrinsic over extrinsic goals.

Among the contributions of this research, a key advancement is the methodology developed to assess the goal content of athletes, based on the latest revision and extension of GCT (see Martela et al., 2019). This constitutes a significant step forward, facilitating the future development of theory in the sports domain. Additionally, encouraging athletes to express their goals in their own words not only helps to avoid the “now that you mention it” effect, which can arise from presenting predefined items (Urdan & Mestas, 2006), but also brings us closer to their reality and provides valuable information for designing meaningful interventions.

From an applied perspective, this study emphasizes the importance of both promoting intrinsic goals and diminishing extrinsic goals during sport practice. There is evidence that social contexts have an important role in the foundation and development of one’s individual intrinsic or extrinsic goals (e.g., Kasser, 2002; Kasser et al., 1995). If the existing environment is supportive and nurtures athletes’ needs, it can facilitate the development of intrinsic goals, but if the needs are frustrated by the social context, individuals will be more prone to adopt need substitutes, such as extrinsic goals (Ryan & Deci, 2017). In this way, sports professionals (e.g., sports psychologists, coaches, and fitness trainers) could, through the motivational climates they create, encourage athletes to set and pursue intrinsic goals, such as goals to do with personal growth or mastery, and to reduce “winning” goals or other common extrinsic goals. This should not be confused with promoting more self-determined reasons for pursuing goals, which is also very relevant. To this end, it is essential for sport professionals and researchers to distinguish between the content of the goals (the “what”) and the reason for pursuing them (the “why”). Both aspects should be considered when conducting research on goal pursuit as well as when working with athletes.

## Figures and Tables

**Table 1:** Inter-rater agreement, frequency distribution, and percentages for individual goal categories, subcategories, and controllability.

	Inter-Rater Agreement				Frequency Distribution and Percentages
	Kappa	*p* Value	LL 95% CI	UL 95% CI	*n* = 414 (%)
Categories					
Intrinsic	0.74	0.001	0.67	0.81	256 (61.8)
Extrinsic	0.79	0.001	0.72	0.86	130 (31.4)
Ambiguous	0.37	0.001	0.30	0.44	28 (6.8)
					414 (100)
Intrinsic goal subcategories					
Social affiliation	0.77	0.001	0.69	0.84	33 (12.8)
Self-expression	0.78	0.001	0.70	0.85	41 (16)
Community contribution	0.67	0.001	0.59	0.74	1 (0.4)
Personal growth	0.66	0.001	0.58	0.73	5 (2)
Mastery	0.73	0.001	0.65	0.80	79 (30.9)
Psychological and physical health	0.89	0.001	0.82	0.97	14 (5.5)
Multiple intrinsic goals	0.49	0.001	0.41	0.56	83 (32.4)
					256 (100)
Extrinsic goal subcategories					
Financial success	1.0	0.001	0.93	1.07	3 (2.3)
Social recognition/Popularity	0.60	0.001	0.52	0.67	2 (1.5)
Multiple extrinsic goals	0.83	0.001	0.76	0.91	125 (96.2)
					130 (100)
Controllability					
Controllable	0.81	0.001	0.72	0.90	173 (41.8)
Uncontrollable	0.81	0.001	0.72	0.90	241 (58.2)
					414 (100)

**Table 2:** Descriptive statistics, reliability, and bivariate correlations.

	Range	M	SD	α	1	2	3	4	5	6
1. Self-efficacy for goal attainment	1–7	5.93	1.00	-	-					
2. Autonomous goal motives	1–7	6.32	0.76	0.70	0.27 **	-				
3. Controlled goal motives	1–7	2.38	1.29	0.72	−0.08	−0.19 **	-			
4. Goal difficulty	1–7	4.89	1.40	0.87	−0.35 **	0.08	0.04	-		
5. Goal-directed effort	1–7	5.90	0.99	0.91	0.12 *	0.42 **	−0.09	0.19 **	-	
6. Vitality	1–7	4.95	1.13	0.87	0.16 **	0.14 **	−0.05	0.01	0.14 **	-
7. Physical and emotional exhaustion	1–5	2.17	0.83	0.88	−0.20 **	−0.23 **	0.24 **	0.03	−0.16 **	−0.22 **

* *p* < 0.05. ** *p* < 0.01.

**Table 3:** Means, standard errors, and analyses of variance in goal-related variables and well and ill-being indicators.

Measure	Intrinsic Goal		Extrinsic Goal		F(1, 383)	η^2^
	*M*	*SE*	*M*	*SE*		
Self-efficacy for goal attainment	6.03	0.06	5.74	0.09	5.99 *	0.02
Autonomous goal motives	6.32	0.05	6.32	0.07	0.01	0.01
Controlled goal motives	2.39	0.08	2.35	0.11	0.19	0.01
Goal difficulty	4.57	0.08	5.51	0.12	39.84 **	0.10
Goal-directed effort	5.88	0.06	5.96	0.09	0.41	0.01
Vitality	4.95	0.07	4.95	0.10	0.03	0.01
Physical and emotional exhaustion	2.15	0.05	2.22	0.07	0.73	0.01

* *p* < 0.05. ** *p* < 0.01.

## Data Availability

The datasets presented in this article are not readily available because additional studies are using the dataset. Requests to access the datasets should be directed to natalia.martinez@uv.es.

## Appendix A

This document includes the detailed definitions of each category or subcategory based on the GCT, which allow for the classification of goals.

- Categories:
  ○ Intrinsic goal: It pursues the individual’s personal growth and personal and social development. It is inherently fulfilling and reflects innate human tendencies towards growth;
  ○ Extrinsic goal: It reflects the individual’s desire to achieve external indicators of value, such as being recognized, looking good, or being wealthy. It depends on the approval or judgment of others;
  ○ Ambiguous: It does not fit into one of these categories, or there is not enough information for it to be included in one of the categories.
- Controllability:
  ○ Controllable: It depends on internal factors (e.g., effort) that are under the voluntary control of the athlete.
  ○ Uncontrollable: It depends on external factors (e.g., difficulty of the task or luck) that cannot be controlled by the athlete.
- Intrinsic goals subcategories:
  ○ Social affiliation: Form close/meaningful links with others through sport;
  ○ Self-expression: Freely pursue self-interests and passions;
  ○ Community contribution: To contribute and help the community through activism or generosity;
  ○ Personal growth: To achieve psychological growth, personal development, or self-acceptance;
  ○ Mastery: To seek the acquisition or development of high-level skills and capabilities or mastery of the task;
  ○ Health: To improve psychological and physical health or fitness;
  ○ Multiple intrinsic goals: Goal that reflects a mix of two or more intrinsic subcategories.
- Extrinsic goals subcategories:
  ○ Social adherence: To be included in a social group and to avoid social exclusion;
  ○ Financial success: To possess money or material goods;
  ○ Image: To improve image or physical appearance;
  ○ Power: To have control or dominance over others;
  ○ Social recognition/Popularity: To gain recognition and admiration from other people or to be famous;
  ○ Superiority: To perform better than other people, social comparison;
  ○ Multiple extrinsic goals: Goal that reflects a mix of two or more extrinsic subcategories.
